# Responses of the Leaf Characteristics of *Nymphoides peltata* to a Water Depth Gradient in the Qionghai Lake, Western Sichuan Plateau, China

**DOI:** 10.3390/plants14060919

**Published:** 2025-03-14

**Authors:** Qun Li, Lan Chen, Yumei Qiu, Xiaoyan Li, Zhe Nan, Shulin Yao, Zhenghong Chen, Yuhan Zhang, Chengzhang Zhao

**Affiliations:** 1 College of Resources and Environment, Xichang University, Xichang 615013, China; 19119164428@163.com (L.C.); 18011029242@163.com (Y.Q.); kerlion@126.com (Z.N.); 19882027523@163.com (S.Y.); 18483153072@163.com (Z.C.); 13518397742@163.com (Y.Z.); 2 School of Life Sciences, Fudan University, Shanghai 200433, China; 3 College of Animal Science, Xichang University, Xichang 615013, China; xiaoyanli_315@163.com; 4 College of Geography and Environmental Science, Northwest Normal University, Lanzhou 730070, China; zhaocz1710@163.com

**Keywords:** leaf area, leaf thickness, leaf petiole diameter, phenotypic plasticity, aquatic plant

## Abstract

The correlations between leaf traits of plants with floating leaves and the responses of these traits to changes in water depth can be used to explore the ecological adaptation strategies of aquatic plants. However, few studies have investigated the covariation and correlation of leaf petiole and leaf morphological indices of aquatic plants along natural water depth gradients. Three plots were established along a water depth gradient: plot I (shallow water, with a water depth ranging from 0 to 20 cm), plot II (medium water, with a water depth ranging from 20 to 40 cm), and plot III (deep water, with a water level ranging from 40 to 60 cm). The floating plant *Nymphoides peltata* (S. G. Gmel.) Kuntze was studied in the Qionghai National Wetland Park, Sichuan Province, China. The results showed that *N. peltata* had large, thin leaves and short, thin leaf petioles in plot I; the leaf petiole and leaf traits were opposite of those in Plot III. In the three plots, leaf petiole length and leaf petiole diameter were significantly negatively correlated with leaf area, leaf circumference, leaf length, and leaf width (*p* < 0.05). *N. peltata* can maintain normal growth, survival, and reproduction in heterogeneous habitats with different water depths by altering its leaf morphological characteristics in a timely manner. This study is helpful for understanding the mechanism of phenotypic plasticity in aquatic plants with floating foliage in heterogeneous environments and provides a scientific basis for the management of aquatic plants in wetlands.

## 1. Introduction

Plant functional traits are a series of morphological, physiological, structural, and other attributes that affect plant performance or adaptability [1] and can significantly affect ecosystem functions and reflect plant response strategies to environmental changes [2,3]. Leaves are sensitive to environmental changes and have strong plasticity [4]. They are the primary location of energy conversion and main organ through which plants obtain carbon via photosynthesis. Leaf phenotypic characteristics are regulated by both genetic factors and environmental factors [5,6]. The adaptive changes in leaf area, leaf thickness and chlorophyll in heterogeneous environments are the result of long-term adaptations of plant growth requirements to spatial heterogeneity or environmental changes [7]. Water level variations (WLVs) determine the distribution patterns of aquatic macrophytes [8,9]. Aquatic plants showed high phenotypic plasticity in response to water level changes [10,11]. It is mainly reflected in morphology adjustment, biomass reallocation, and changes in the content of photosynthesis pigments and root traits [12,13,14,15]. For example, when the water level rises, aquatic plants can transfer photosynthetic products to plant stem and leaf parts and connecting organs, resulting in the elongation of stems or leaves and enhancing the acquisition and utilization of O_2_, CO_2_ and light resources while reducing the allocation of resources to root biomass [12]. The leaf petiole supports the leaves, arranges the leaves to receive more sunlight in a certain space, and connects the water and nutrient transport between the leaves and stems. Meanwhile, the length of the leaf petiole determines the size of the leaves and the ability of the leaves to receive light resources [16]. The stem is an important organ that transports nutrients between roots and leaves and provides physical support [17]. The stem connects the leaves and roots, provides physical support for both leaves and roots, and plays a role in resource transport, thus supporting plant growth to varying degrees under different environmental influences. The leaf petiole and stems of aquatic plants elongate with increasing water levels and elongate less with decreasing water levels [13,14,18]. The resources required for the growth of roots and leaves are adjusted rapidly with changes in the environment to reduce nutrient waste [15] and improve the adaptability of plants to heterogeneous environments. Leaf petioles and leaves are important organs for water and nutrient transport and resource acquisition in plants, and they are closely related to plant survival, growth, and function. The growth patterns of these organs directly determine the biomass allocation pattern and photosynthetic efficiency of plants and have an important impact on the carbon acquisition and light interception ability of plants [17].

Wetlands are complex and unique ecosystems that occur in the transition zone between water and land and play an important role in maintaining the functionality of biodiversity and the integrity of the biological chain; these areas also provide many environmental benefits. Lake wetlands are affected by precipitation, direct and indirect runoff from adjacent basins, surface evaporation, and interactions between lakes and groundwater levels, and their ecohydrological processes are very complex [19,20]. Water level change is an important environmental factor affecting aquatic plants and can directly affect water temperature, light availability, transparency, etc. [21]. Therefore, changes in the water level affect the distribution patterns and resource acquisition abilities of aquatic plant communities and populations [22] and alter morphological and physiological processes such as plant growth and biomass allocation. Therefore, investigating the morphological characteristics of aquatic plants and their correlation with water level changes is very important for understanding the mechanism of phenotypic plasticity and ecological adaptation strategies of aquatic plants in heterogeneous habitats. Previous studies on aquatic plants have focused mainly on the influence of water level changes on aquatic plant morphology and physiology [23,24,25,26,27], the influence of light on aquatic plant phenotypes [28], the influence of aquatic plants on sediment and bacterial community structure [29,30], and the role of aquatic plants in ecological restoration [31]. Most of these studies involve controlled indoor experiments. However, there are few studies on the covariation and correlation of leaf petiole and leaf morphology in aquatic plants with natural changes in the water level.

*Nymphoides peltata* (S. G. Gmel.) Kuntze is a perennial floating aquatic plant belonging to genus *Menyanthaceae*. It usually lives in groups and forms monodominant communities, mostly in the transitional zone between emergent plants and open water. In recent years, research on *N. peltata* in China and other countries has focused mainly on the influence of competition on resource allocation [32], clonal growth [33], and the absorption of environmental pollutants [34]. Studies have also investigated the effects of invasion by alien species on *N. peltata* [35] and adaptive changes and photosynthetic characteristics of *N. peltata* under stress [36]. However, there are few studies on the responses of the leaf petiole and leaf traits of *N. peltata* to environmental factors and the mechanism of adaptation.

Therefore, we hypothesized that (1) the leaf petiole and leaf traits of *N. peltata* change significantly with changes in the water level gradient and (2) there is a certain correlation between the leaf petiole and leaf characteristics of *N. peltata*. This study aimed to explore the response strategies of the leaf petiole and leaf morphological traits of aquatic plants to differences in water depth and to understand the mechanism of ecological adaptation of aquatic plants at different points along a water depth gradient to provide a theoretical basis for the protection, development, and utilization of wetland aquatic plants.

## 2. Materials and Methods

### 2.1. Study Sites and Sampling

**Figure 1:**
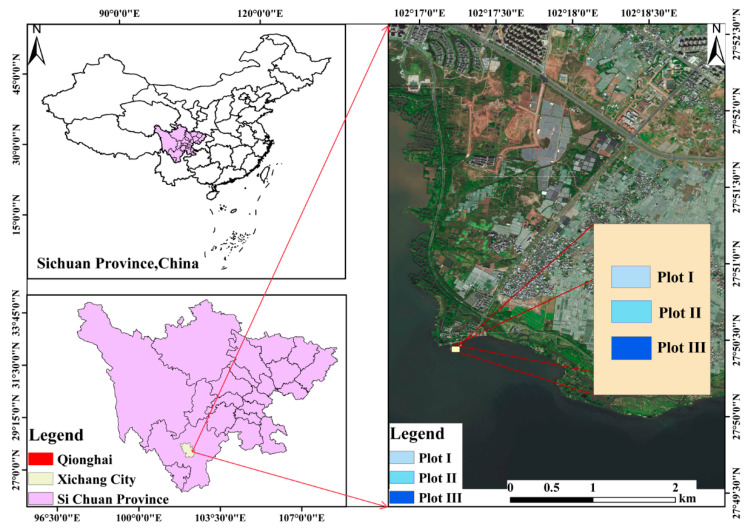
Study area and locations of the plots (plotted by software, version 10.8, Arcgis).

The study site is located in Qionghai National Wetland Park (102°15′~102°18′ E, 27°42′~27°55′ N; Figure 1), Xichang city, Liangshan Yi Autonomous Prefecture, Sichuan Province, China. It is located along the eastern margin of the Hengduan Mountain area of the Qinghai–Tibet Plateau, in the southwestern subtropical plateau mountain area, at the northeastern foot of Lushan Mountain, and on the north side of Luoji Mountain. The elevations of the study site range from 1500 m to 3500 m. The study area is characterized by a humid subtropical plateau monsoon climate with warm winters, cool summers, and distinct dry and wet seasons [37]. The annual average temperature is 17 °C, the annual precipitation is 1004.3 mm, the annual average evaporation is 1945 mm, the annual average sunshine duration is 2431.4 h, and the annual average wind speed is 15 m/s.

With a circumference of 37.4 km and an area of 31.4 km^2^, Qionghai Lake is located in the Yalong River system of the Yangtze River Basin, with an average depth of 10.95 m and a maximum depth of 18.32 m. It is a plateau freshwater lake located at the source of the Haihe River, a tributary of the Anning River, with a water storage capacity of 289 million m^3^. It is an important resource for Xichang and serves as an ecological barrier in the upper reaches of the Yangtze River. A total of 22 species of aquatic plants belonging to 15 families and 18 genera are found in Qionghai Lake. The dominant plant species are *N. peltata*, *Phyllostachys heteroclada* Oliv., *Phragmites australis* (Cav.) Trin. ex Steud, *Arundo donax* L., *Acorus calamus* L., *Nelumbo nucifera* Gaertn., *Zizania latifolia* (Griseb.) Turcz. ex Stapf, *Schoenoplectus tabernaemontani* (C. C. Gmel.) Palla, *Iris tectorum* Maxim, *Eleocharis dulcis* (Burm. f.) Trin. ex Hensch, *Canna glauca* L., *Sagittaria trifolia* L., *Nymphaea tetragona* Georgi, *Trapa incisa* Siebold & Zucc, *Alternanthera philoxeroides* (Mart.) Griseb, *Lemna minor* L., *Pontederia crassipes* Mart., *Ceratophyllum demersum* L., *Potamogeton wrightii* Morong, *Vallisneria natans* (Lour.) H. Hara, and *Potamogeton crispus* L.

### 2.2. Experimental Methods and Design

From 7 to 10 April 2023, the weather was fine, there was no significant precipitation, and the water level in the study area was relatively stable. Based on many previous investigations of aquatic plant communities in Qionghai Lake and their arrival, a study area measuring 200 m wide and 100 m long was placed along the shoreline of the lake and extending towards the center of the lake. The aquatic plant *N. peltata* was the dominant species in this study area, which had no obvious human interference. Based on the change in the water level, three water depth gradients were established: one in the shallow water area (I, water depth 0~20 cm, 0~20 m from the shore), one in the middle water area (II, water depth 20~40 cm, water depth 20~30 m from the shore), and one in the deep-water area (III, water depth 40~60 cm, water depth 30~50 m from the shore).

First, according to the gradient, five 1 m × 1 m quadrats were placed from shallow water to deep water, totaling 15 quadrats. Then, the stand characteristics of *N. peltata* were investigated in each sample plot, the density and coverage of *N. peltate* along each gradient were recorded, and photographs were taken. Second, longitude, latitude, and elevation were recorded by GPS (X28, China, YiLi, Chengdu). Meanwhile, the water samples were taken at each sample site with a water sampler (CS-100, China, Airel, Suzhou) and each water depth gradient were repeated 5 times, totaling 15 water samples. Next, the transparency of the water in areas with *N. peltata* was measured by Secchi Disk Method (SD20, China, HongPu, Ningbo). During the measurement process, the disk is lowered into the water with a sling, until the disk is almost invisible from above, and the length of the submerged part of the sling is measured. The measurements were repeated five times, and the average value was calculated. Finally, *N. peltata* in each sample plot was sampled with a grab bucket (50 × 38 cm) specially designed for aquatic plants. Each plot was sampled five times, and the collected *N. peltata* were placed in a numbered net. The soil and debris attached to the surface of the plants were removed using a nearby water source, and the plants were packed into self-sealing bags and numbered before being transported to the laboratory for measurement of biomass and other leaf petiole and leaf properties.

#### 2.2.1. Measurement of *N. peltata* Leaf Morphological Characteristics

In the laboratory, 6 *N. peltata* plants with relatively complete rhizomes and leaves were selected from each quadrat. Then, 2–3 healthy and intact leaves were selected from each *N. peltata* plant for investigation of petiole and other leaf traits of *N. peltata*. Height and leaf petiole length were measured with a ruler after the plants were rinsed and blotted dry to remove the surface water. A Vernier caliper (accurate to 0.01 mm) was used to measure the leaf petiole diameter and leaf thickness. The center of each leaf was used for measuring leaf thickness, avoiding veins when possible. Three replicates were performed for each leaf trait measurement, and the average was calculated. The chlorophyll content of fresh *N. peltata* leaves was measured by a portable chlorophyll meter (SPAD-502, Minolta, Osaka, Japan) [38]; this process was repeated 3 times, and the average value of these measurements was obtained. The leaf area, leaf circumference, leaf length, and leaf width of *N. peltata* were measured and recorded (CI-202, Walz, Camas, WA, USA) [39], with three replicates per leaf, and the average was calculated. The fresh leaf weight was measured 3 times using an electronic balance (accurate to 0.0001 g), and the average value was calculated. Finally, the *N. peltata* samples were placed in envelopes with numbers corresponding to the sample plots and baked in an oven at 105 °C for 12 h, after which leaf dry weight and leaf petiole dry weight were obtained. The SLA was expressed as the ratio of LA to leaf dry weight [22].

#### 2.2.2. Measurement of Water Salinity and pH

The salinity of the water samples was measured using the EC method. Within a certain range of concentrations, the salt content of a solution is positively correlated with its electrical conductivity, so the electrical conductivity can be used to represent the water salt content [40]. A portable conductivity meter (DDS-11C, Shanghai Lei Magnetic Instrument Factory, Shanghai, China) and portable soil pH meter (ST3100, Ohaus Instruments Co., Ltd., Shanghai, China) were used to measure the EC and pH of the leachate, respectively [39]. Three replicates were performed for each sample, and the average was calculated.

### 2.3. Statistical Analysis

Microsoft Excel 2019 was used to organize all the experimental data. The population trait and water environmental data for the three gradients were statistically analyzed. One-way ANOVA was used to compare the leaf traits and population traits of *N. peltata* among the different habitats (α = 0.05). After all the data were standardized using the statistical option in SPSS (version 22.0, SPSS, Chicago, IL, USA), redundancy analysis (RDA, type II scaling) was performed with CANOCO4.5 software to determine the responses of the most significant trait variables to environmental factors [41]. Then, Origin 2022 software was used for correlation analysis, with the significance level set at 0.05. The plasticity indices of the leaves of *N. peltata* can reflect the potential adaptability of the plants to the environment. The plasticity indices of the leaves of *N. peltata* were calculated as follows: the (maximum − minimum)/maximum value of the leaf character index of a given plant along three water level gradients. The mean and standard error (SE) of five replicates were obtained for each measurement.

## 3. Results

### 3.1. Physical and Chemical Characteristics of Water

**Figure 2:**
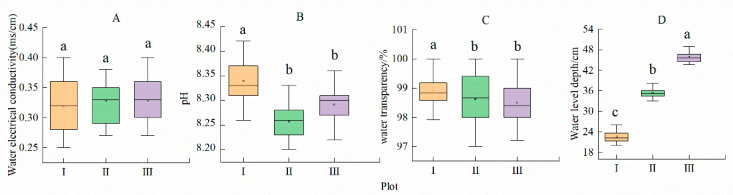
Characteristics of environmental factors affecting *Nymphoides peltata*. Different lowercase letters indicate significant differences among plots (*p* < 0.05). n = 15. I—shallow water area; II—middle water area; III—deep-water area. (**A**) Water electrical conductivity in each plot; (**B**) Water pH in each plot; (**C**) Water transparency in each plot; (**D**) Water level depth in each plot.

The significant changes were found at different water depths (*p* < 0.05, Figure 2, Table S1). The water depth increased 1.89-fold from the shallow water area (plot I) to the deep-water area (plot III) (Figure 2D). The pH and water transparency did not change significantly from plot I to plot III (Figure 2B,C; *p* > 0.05). With the habitat conditions changing from plot I to plot III, the pH and water transparency decreased by only 0.43% and 0.83%, respectively. In addition, there was no significant change in the electrical conductivity of the water (Figure 2A; *p* > 0.05).

### 3.2. Stand Characteristics of Nymphoides peltata

**Figure 3:**
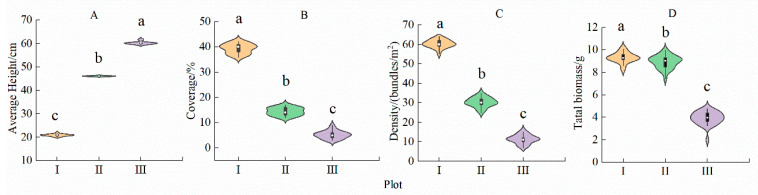
The stand characteristics of *Nymphoides peltata* in each plot. Different lowercase letters indicate significant differences among plots (*p* < 0.05). n = 30. I—shallow water area; II—middle water area; III—deep-water area. (**A**) Average height of *N. peltata* population in each plot; (**B**) Coverage of *N. peltata* in each plot; (**C**) Density of *N. peltata* population in each plot; (**D**) Total biomass of *N. peltata* population in each plot.

The stand characteristics of *N. peltata* changed significantly with the increasing water depth (*p* < 0.05, Figure 3, Table S2). The average height was lowest in the shallow water area (plot I) and the largest in plot III (Figure 3A). The coverage, density, and total biomass were greatest in plot I and lowest in plot III (Figure 3B–D). With the change in the habitat from plot I to plot III, the average height of the *N. peltata* population increased (Figure 3A) 1.87-fold, while the coverage, density, and total biomass showed decreasing trends, and decreased by 80.97%, 86.95%, and 58.00%, respectively (Figure 3B–D; Table S2).

### 3.3. Main Characteristics (Leaf and Leaf Petiole) of Nymphoides peltata Along a Water Depth Gradient

The main morphological characteristics (leaf and leaf petiole) of *N. peltata* significantly changed under different habitat conditions (*p* < 0.05, Table 1). The leaf area, leaf perimeter, leaf length, leaf width, and fresh leaf weight were largest in the shallow water area (plot I) and smallest in the deep-water area (plot III). The leaf petiole length and chlorophyll content were lowest in plot I and greatest in plot III. Leaf thickness was lowest in plot I and largest in the middle water area (plot II). The leaf petiole diameter was lowest in plot II and greatest in plot III. With the change in habitat from plot I to plot III, the leaf area, leaf perimeter, leaf length, leaf width, fresh leaf weight, and specific leaf area decreased by 33.43%, 11.55%, 13.42%, 22.43%, 67.02%, and 49.58%, respectively. The leaf petiole length and chlorophyll content exhibited increasing trends and increased by 98.96% and 10.05%, respectively. The leaf thickness, leaf dry weight, leaf petiole diameter, and leaf petiole dry weight first increased, then decreased, and ultimately increased by 92.86%, 40%, 35.29%, and 66.67%. The plasticity index of the leaf and leaf petiole morphological characteristics of *Nymphoides peltata* was leaf thickness, fresh leaf weight, leaf petiole dry weight, specific leaf area, leaf petiole length, leaf dry weight, leaf petiole diameter, leaf area, leaf wide, leaf length, leaf perimeter, and CHL, in order from large to small.

### 3.4. Results of the RDA of the Leaf Traits of and Environmental Factors Affecting Nymphoides peltata

**Figure 4:**
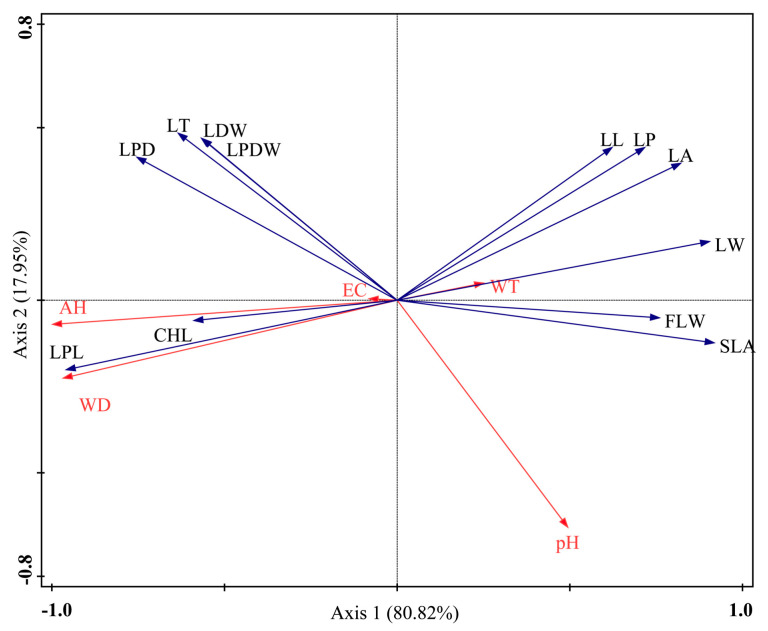
RDA ordination of functional traits of and environmental factors affecting *Nymphoides peltata*. The blue arrow represents the leaf traits of *N. peltata*, and the red arrow represents the environmental factor. EC—soil electrical conductivity; WT—water transparency; WD—water depth; AH—average height; LT—leaf thickness; LA—leaf area; SLA—specific leaf area; LP—leaf perimeter; LL—leaf length; LW—leaf width; LDW—leaf dry weight; LPL—leaf petiole length; FLW—fresh leaf weight; LPD—leaf petiole diameter; LPDW—leaf petiole dry weight; CHL—chlorophyll content.

CANOCO4.5 software was used to conduct canonical correspondence analysis of the morphological characteristics and biomass allocation of *N. peltata* in the study area, and the relationships between the axes and environmental factors, as well as the characteristic values of each axis, were obtained (Table 2). The eigenvalues for axes 1 and 2 were 0.56 and 0.12, respectively, and the correlation coefficients between the functional traits and environmental factors were 0.98 and 0.70, respectively. Axes 1 and 2 explained 80.82% and 17.95% of the ecological variation, respectively (Figure 4), indicating that these two axes explained most of the sequencing information. Therefore, the information explained by axes 1 and 2 was used to analyze the relationships between plant functional traits and environmental factors.

In Figure 4, each blue arrow represents a plant functional trait, each red arrow represents an environmental factor, and the line between the dotted arrow and the origin represents the impact of the environmental factor on the plant functional traits. The longer the line is, the greater the impact. The angle between the arrow and the axis indicates the correlation between the environmental factor and the axis. The smaller the angle is, the stronger the correlation. According to the RDA ordination, average height (AH) showed the greatest correlation with the first axis, followed by water depth (WD), pH, and water transparency (WT), with correlation coefficients of −0.98, −0.95, 0.48, and 0.25, respectively (Table 2, Figure 4). WD, AH, and WT were less strongly correlated with the second axis, with correlation coefficients of −0.16, −0.05, and −0.03, respectively. Electrical conductivity (EC) was less strongly correlated with the first axis and second axis, with correlation coefficients of −0.08 and 0.003, respectively (Table 2, Figure 4). The correlation between pH and axis 1 was greater than that between pH and axis 2, with correlation coefficients of 0.48 and −0.46, but pH had less of an effect on the morphology of the leaves.

Figure 4 shows that WD gradually decreased from left to right along RDA axis 1, and WT and pH gradually increased from left to right along RDA axis 1. The leaf petiole length (LPL), leaf thickness (LT), leaf petiole diameter (LPD), leaf dry weight (LDW), and leaf petiole dry weight (LPDW) on the left side of axis 1 were mainly affected by WD and were negatively correlated with WT and pH. However, leaf length (LL), leaf perimeter (LP), leaf area (LA), leaf width (LW), specific leaf area (SLA), and fresh leaf weight (FLW) were mainly affected by WT and pH and were negatively correlated with WD. The CHL of *N. peltata* leaves were located closest to the origin of the sorting axis, indicating that they were less affected by environmental factors. Among these traits, LT, LPL, LPD, LDW, LPDW, LA, LP, SLA, LL, LW, and FLW were strongly affected by environmental factors.

### 3.5. Correlation Analysis of the Leaf Characteristics of Nymphoides peltata

**Figure 5:**
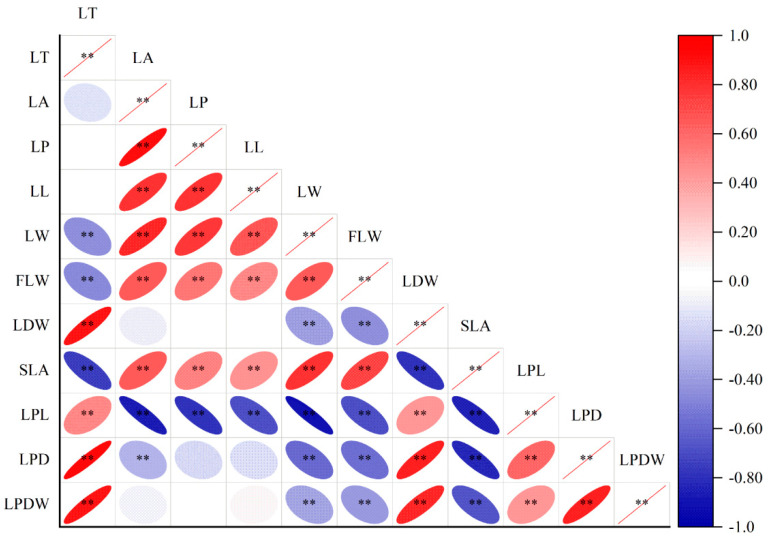
Correlation analysis between leaf traits of *Nymphoides peltata*. ** *p* < 0.01 (significant at the 0.01 level bilaterally; the null hypothesis is rejected at the 99% confidence level, and the sample has a linear correlation). LT—leaf thickness; LA—leaf area; SLA—specific leaf area; LP—leaf perimeter; LL—leaf length; LW—leaf width; LDW—leaf dry weight; LPL—leaf petiole length; LPD—leaf petiole diameter; LPDW—leaf petiole diameter; FLW—fresh leaf weight. Blue represents a positive correlation between leaf traits, and red represents a negative correlation between leaf traits. The deeper the color is, the more significant the correlation. The lighter the color is, the weaker the correlation.

Based on the results described in Section 3.4, the environmental factors that had no significant influence on the stem or leaf traits of *N. peltata* and the leaf traits that were not significantly influenced by the environment were eliminated, and the correlation between the leaf traits of *N. peltata* was analyzed by Pearson correlation. The Pearson correlation analysis results are shown in Figure 5 (Table S3). There was a highly significant negative correlation (*p* < 0.01) between leaf petiole length and leaf area (R^2^ = −0.88), leaf perimeter (R^2^ = −0.79), leaf length (R^2^ = −0.68), leaf width (R^2^ = −0.91), and fresh leaf weight (R^2^ = −0.70); between leaf petiole diameter and leaf area (R^2^ = −0.30), leaf width (R^2^ = −0.60), and fresh leaf weight (R^2^ = −0.57); and between leaf petiole dry weight and leaf width (R^2^ = −0.37), and fresh leaf weight (R^2^ = −0.42). There was also a highly significant positive correlation between leaf petiole length, leaf petiole diameter, leaf petiole dry weight, and leaf thickness (R^2^ = 0.49), and leaf dry weight (R^2^ = 0.43) in all three plots. In addition, there was no significant correlation (*p* > 0.05) between leaf thickness and leaf area (R^2^ = −0.14), leaf perimeter (R^2^ = −0.01), or leaf length (R^2^ = 0.01).

## 4. Discussion

Leaves are the main organs of photosynthesis and gas and water exchange with the atmosphere [42]. Leaf functional traits are physiological and structural characteristics of plants that have evolved due to adaptation to the environment. Leaf phenotypic characteristics are a direct reflection of environmental changes; these characteristics are manifested in changes in different leaf traits (such as leaf size and leaf shape) and are an important part of plant functional traits [5,6,43,44]. With increasing water depth, *N. peltata* tended to form small and thick leaves and thick and long leaf petioles (Table 1). The leaf petiole length and leaf petiole diameter of *N. peltata* in the three plots were negatively correlated with leaf area, leaf circumference, leaf length, and leaf width (*p* < 0.01, Figure 2). Differences in the water environment in the Qionghai wetland have led to changes in the environment and resource conditions required for the survival of *N. peltata*. By adjusting its leaf phenotypic traits, *N. peltata* leaves became suitable for its habitat. The results showed that *N. peltata* had strong tolerance to and phenotypic plasticity in heterogeneous water environments.

### 4.1. Response of N. peltata Leaf Traits to Differences in Water Depth in Shallow Water Areas

Leaf traits are not only closely related to the resource acquisition and utilization efficiency of plants but also reflect the survival strategies of plants to adopt differentiated leaf building models to maximize carbon assimilation products [4,45]. In shallow water areas, the water level was the lowest (0–20 cm) (Figure 2, Table S1). The coverage and density of *N. peltata* were the greatest (Figure 3, Table S2). In the shallow water area, *N. peltata* tends to have large, thin leaves and short, thin leaf petioles (Table 1). The main reasons for these traits are as follows. (1) The coverage and density of *N. peltata* were greatest in the shallow water, and intraspecific competition was strong. Given the limited resources, the development of thin and large leaves with high SLA was conducive to increasing the area of light exposure and expanding the surface area for contact with the air, and improving the photosynthetic efficiency [46]. Moreover, large and thin leaves can shorten the path of CO_2_ and water from stomata to chloroplasts, which is conducive to light transmission and light energy absorption [47,48], thus increasing the utilization capacity of light resources by *N. peltata*. (2) The water depth was lowest in plot I, and the water level fluctuated greatly [49]. The relatively thick leaf petioles are not conducive to adaptation to water environments with frequent fluctuations, and thin leaf petioles have strong resistance to mechanical interference [14]. Therefore, *N. peltata* developed short and thin leaf petioles in this habitat (Table 1), and leaf petiole length and leaf petiole diameter were significantly negatively correlated with leaf area, leaf circumference, leaf length, and leaf width (*p* < 0.01, Figure 5, Table S3). The results showed that in shallow water, *N. peltata* formed thin and short leaf petioles by adjusting the morphological structures of the leaf petioles and leaves and increasing the investment in leaf resources (Table 1); this adjustment reflected the strong phenotypic plasticity of the leaf petioles and leaves of this floating foliage species. This is consistent with the conclusion of aquatic *Ludwigia grandiflora* (Michx.) Greuter & Burdet reported by Scremin-Dias et al. (2023) [27].

### 4.2. Response of the Leaf Traits of the Floating Heart-Growing N. peltata to Differences in Water Depth in Deep-Water Areas

Plant leaves are exposed to the environment and are thus the most sensitive parts of aquatic plants to environmental changes, and the leaf is the first to respond to environmental changes [50,51]. During the experiment, at plot III, the water level was the highest (40–60 cm) (Figure 2, Table S2). In this habitat, the coverage and density of *N. peltata* were the lowest (Table 1). *N. peltata* tends to have small, thick leaves and long, thick leaf petioles (Table 1). The main reasons for these traits are as follows. In deep water, *N. peltata* has the lowest coverage and density and the weakest interspecific competition. When resources are limited, *N. peltata* can obtain sufficient light resources without a large leaf area. Moreover, small leaves can be well-adapted to the high-light environment in the deep-water area due to the loss of intraspecial shade [39,52], while thick leaves with longer palmar cells and more cell layers reduce the penetration of light radiation, prevent injury by intense light, and improve leaf water use efficiency and light energy use [53]. The use of limited photosynthetic carbon assimilation products in the construction of support structures is helpful for coping with the external pressure from increasing water depth, increasing the mechanical support of *N. peltata*, and improving the adaptability of *N. peltata* to the water environment. Therefore, *N. peltata* developed leaves with small areas but relatively high thicknesses and long and thick leaf petioles (Table 1). Leaf petiole length and leaf petiole diameter were significantly negatively correlated with leaf area (*p* < 0.01; Figure 5, Table S3). The results showed that with increasing water level, *N. peltata* adjusted the allocation of leaf petiole and leaf resources in a timely manner (Table 1), formed small and thick leaves and long and thick leaf petiole and leaf components suitable for the habitat, and improved its adaptability to the aquatic environment, reflecting the strong ecological adaptability of this species. This finding indicates significant differences relative to the leaf phenotypic plasticity of the wetland plants *Saussurea salsa* and *Phragmites australis* reported by Li Qun et al. (2019, 2021, 2022) [22,39,41] in flooded environments. The studies found that these two plant species in the lakeshore area adopted larger and thinner leaves, which differ from the leaf construction patterns of *N. peltata* observed in this study. Moreover, Yu and Yu (2011) [23] reported that the size of *Nymphoides peltata* (S. G. Gmel.) Kuntze leaves and leaf petioles increased rapidly along a water level gradient, leading to a consistent conclusion, namely, that plants with floating leaves adapt to fluctuations in the water environment.

To ensure that the physiological needs of plants are met, plant leaf traits are optimized to achieve a balance between survival and growth [54]. The water depths of the *N. peltata* habitats varied between the area with shallow water (plot I) and that with deep water (plot III), and the leaf area, leaf perimeter, leaf length, leaf width, leaf fresh weight, leaf petiole length, and leaf petiole diameter all varied between plot I and plot III (Table 1). In the presence of limited resources, *N. peltata* has a moderate leaf size and support structure (Table 1) to maximize the light energy utilization and capture ability of leaves. Leaf morphological characteristics are adjusted in a timely manner, allowing plants to adapt well to the environment and maintain normal growth and reproductive strategies.

## 5. Conclusions

To adapt to different environmental conditions, *N. peltata* has large, thin leaves and thin and short leaf petioles. These traits allow the plants to maximize the utilization of space and optimize the allocation of internal resources in shallow water areas. In deep-water areas within our study site, *N. peltata* reduces resource allocation to leaves while increasing investment in petioles, constructing smaller and thicker leaves and longer, thicker petioles to adapt to environmental changes. The patterns of leaf development with the change in the water level is the result of the long-term adaptation of aquatic plants to environmental changes and reflects an ecological adaptation strategy of floating aquatic plants in wetlands. This study explored the mechanism of phenotypic plasticity of the leaf morphological traits of aquatic plants only in response to water depth at a single study site. The other morphological traits of *N. peltata* (roots, stems, and leaves) also play important roles in responding to changes in aquatic environments. Water N, P, and dissolved oxygen levels also significantly impact the growth and development of aquatic plants, requiring further investigation and research.

## Figures and Tables

**Table 1:** The leaf traits of *Nymphoides peltata* (mean ± SE).

Plot	I	II	III	Plasticity Index
Leaf thickness (mm)	0.14 ± 0.01 c	0.43 ± 0.01 a	0.27 ± 0.01 b	0.67
Leaf area (cm^2^)	14.51 ± 0.04 a	13.61 ± 0.14 b	9.66 ± 0.08 c	0.33
Leaf perimeter (mm)	15.15 ± 0.01 a	15.06 ± 0.05 a	13.40 ± 0.08 b	0.12
Leaf length (mm)	4.47 ± 0.01 a	4.45 ± 0.02 a	3.87 ± 0.06 b	0.13
Leaf width (mm)	4.19 ± 0.02 a	3.70 ± 0.05 b	3.25 ± 0.01 c	0.22
Leaf petiole length (cm)	23.14 ± 0.40 c	35.37 ± 0.29 b	46.04± 0.41 a	0.50
Fresh leaf weight (g)	0.94 ± 0.07 a	0.52 ± 0.02 b	0.31 ± 0.02 c	0.67
Leaf dry weight (g)	0.05 ± 0.001 c	0.09 ± 0.002 a	0.07 ± 0.001 b	0.40
Specific leaf area (cm^2^/g)	284.05 ± 5.42 a	151.88 ± 2.86 b	142.19 ± 3.35 c	0.50
Leaf petiole diameter(mm)	1.02 ± 0.01 c	1.58 ± 0.01 a	1.38 ± 0.01 b	0.35
Leaf petiole dry weight (g)	0.03 ± 0.001 c	0.07 ± 0.001 a	0.05 ± 0.001 b	0.57
Chlorophyll (SPAD)	31.71 ± 0.32 b	34.11± 0.48 a	34.90 ± 0.10 a	0.09

Different lowercase letters in the same row indicate a significant difference among plots (*p* < 0.05; n = 30). I—shallow water area; II,—middle water area; III—deep-water area.

**Table 2:** RDA results of leaf traits and environmental factors of *Nymphoides peltata*.

Statistic		Axis 1	Axis 2	Total Variance
EC		−0.08	0.003	1
pH		0.48	−0.46	
WT		0.25	−0.03	
WD		−0.95	−0.16	
AH		−0.98	−0.05	
Eigenvalues		0.56	0.12	
function traits–environment correlations		0.98	0.70	
Explained variation (cumulative)	of function traits data	55.65	68.02	
	of function traits–environment relation	80.82	98.77	
All eigenvalues				1
Canonical eigenvalues				0.69

EC—soil electrical conductivity; WT—water transparency; WD—water depth; AH—average height.

## Data Availability

Data are contained within the article.

## Supplementary Materials

The following supporting information can be downloaded at: https://www.mdpi.com/article/10.3390/plants14060919/s1. Table S1: The water environment characteristics of each habitat (mean ± SE); Table S2: The population characteristics of *Nymphoides peltata* in each habitat (mean ± SE); Table S3: Correlation analysis between leaf traits of *Nymphoides peltata*.
