# Supplementary material for: Responses of the Leaf Characteristics of Nymphoides peltata to a Water Depth Gradient in the Qionghai Lake, Western Sichuan Plateau, China
Source: Plants (Basel). 2025 Mar 14;14(6):919. doi: 10.3390/plants14060919 (PMC11944560; doi:10.3390/plants14060919)
Supplement: Supplementary file 1 [file plants-14-00919-s001.zip › plants-3438487-supplementary.pdf]

Table S1 The water environment characteristics of each habitat (mean ± SE)

Plot	EC ($ms·cm^{-1}$)	pH	WT (%)	WD (CM)
I	0.32 ± 0.01a	8.34 ± 0.01a	98.88 ± 0.10a	22.61 ± 0.32c
II	0.33 ± 0.01a	8.25 ± 0.01b	98.61 ± 0.15b	35.28 ± 0.24b
III	0.32 ±0.01a	8.27 ± 0.01b	98.45 ± 0.13b	45.99 ± 0.31a

Different lowercase letters in the same column indicate significant differences among plots ($p < 0.05$). n=15. I, The shallow water area; II, The middle water area; III, The deep water area. EC, Electrical conductivity, WT, water transparency; WD, water level depth.

Table S2 The population characteristics of *Nymphoides peltata* in each habitat (mean ± SE)

Plot	AH (cm)	D ($bundles/m^2$)	Coverage (%)	TB (g)
I	20.90 ± 0.09c	60.27 ± 0.39a	39.30 ± 0.33a	9.31 ± 0.08a
II	46.04 ± 0.04b	30.20 ± 0.41b	14.40 ± 0.26b	8.93 ± 0.10b
III	59.97 ± 0.19a	11.47 ± 0.38c	5.13 ± 0.27c	3.91 ± 0.11c

Different lowercase letters in the same column indicate significant differences among plots ($p < 0.05$). n=30. I, The shallow water area; II, The middle water area; III, The deep water area. AH, Average height, D, Density; TB, Total biomass.

Table S3 Correlation analysis between leaf traits of *Nymphoides peltata*

	LT	LA	LP	LL	LW	FLW	LDW	SLA	LPL	LPD	LPDW
LT	1										
LA	-0.137	1									
LP	-0.013	0.906**	1								
LL	0.007	0.791**	0.787**	1							
LW	-0.452**	0.821**	0.760**	0.675**	1						
FLW	-0.475**	0.642**	0.546**	0.488**	0.651**	1					
LDW	0.883**	-0.093	0.029	0.01	-0.393**	-0.451**	1				
SLA	-0.755**	0.651**	0.517**	0.455**	0.790**	0.734**	-0.798**	1			
LPL	0.490**	-0.876**	-0.788**	-0.683**	-0.907**	-0.697**	0.433**	-0.846**	1		
LPD	0.931**	-0.303**	-0.176	-0.15	-0.598**	-0.573**	0.854**	-0.834**	0.613**	1	
LPDW	0.897**	-0.078	0.038	0.061	-0.370**	-0.417**	0.825**	-0.667**	0.423**	0.858**	1

** $p < 0.01$ (significant at the 0.01 level bilaterally; the null hypothesis is rejected at the 99% confidence level, and the sample has a linear correlation). LT, leaf thickness; LA, leaf area; LP, leaf perimeter; LL, leaf length; LW, leaf wide; FLW, fresh leaf weight; SLA, specific leaf area; LDW, leaf dry weight; LPL, leaf petiole length; LPD, leaf petiole diameter; LPDW, leaf petiole diameter; FLW, fresh leaf weight.
